# Long-term clearance from small airways in subjects with ciliary dysfunction

**DOI:** 10.1186/1465-9921-7-79

**Published:** 2006-05-20

**Authors:** Maria Lindström, Rolf Falk, Lena Hjelte, Klas Philipson, Magnus Svartengren

**Affiliations:** 1 Division of Occupational Medicine, Department of Public Health Science, Karolinska Institutet, Stockholm, Sweden; 2 Stockholm CF center, Department of Paediatrics, Karolinska Institutet, Karolinska University Hospital, Huddinge, Stockholm, Sweden; 3 Division of Inhalation Toxicology, Institute of Environmental Medicine, Karolinska Institutet, Stockholm, Sweden; 4 Swedish Radiation Protection Authority (SSI), Stockholm, Sweden

## Abstract

The objective of this study was to investigate if long-term clearance from small airways is dependent on normal ciliary function.

Six young adults with primary ciliary dyskinesia (PCD) inhaled ^111 ^Indium labelled Teflon particles of 4.2 μm geometric and 6.2 μm aerodynamic diameter with an extremely slow inhalation flow, 0.05 L/s. The inhalation method deposits particles mainly in the small conducting airways. Lung retention was measured immediately after inhalation and at four occasions up to 21 days after inhalation. Results were compared with data from ten healthy controls. For additional comparison three of the PCD subjects also inhaled the test particles with normal inhalation flow, 0.5 L/s, providing a more central deposition. The lung retention at 24 h in % of lung deposition (Ret_24_) was higher (p < 0.001) in the PCD subjects, 79 % (95% Confidence Interval, 67.6;90.6), compared to 49 % (42.3;55.5) in the healthy controls. There was a significant clearance after 24 h both in the PCD subjects and in the healthy controls with equivalent clearance. The mean Ret_24 _with slow inhalation flow was 73.9 ± 1.9 % compared to 68.9 ± 7.5 % with normal inhalation flow in the three PCD subjects exposed twice. During day 7–21 the three PCD subjects exposed twice cleared 9 % with normal flow, probably representing predominantly alveolar clearance, compared to 19 % with slow inhalation flow, probably representing mainly small airway clearance.

This study shows that despite ciliary dysfunction, clearance continues in the small airways beyond 24 h. There are apparently additional clearance mechanisms present in the small airways.

## Introduction

Inhaled insoluble particles deposited in the lung are cleared by various clearance mechanisms depending on the site of deposition. Three different compartments within the lung can be distinguished, the large airways including the main lobar and segmental bronchi; the small airways representing mainly the bronchioles; and the alveolar region. These three compartments might also represent three different clearance phases. The majority of particles deposited in the large airways of healthy subjects are eliminated within 24 h by the mucociliary clearance mechanism, and it has long been assumed that any particles remaining in the lung at 24 h represent alveolar clearance [1,2]. This is probably due to the deposition pattern of particles inhaled with normal inhalation flow which give a limited deposition in the small airways. However, previous studies in healthy subjects using a shallow bolus technique as well as studies using extremely slow inhalation flow (0.05 L/s) and 6 μm particles, resulting in particles deposited in the small airways, show that a considerable fraction of particles may be retained after 24 h [3-5]. This slow clearance phase, probably representing the small airways, continues and is shown to be faster than the alveolar clearance but much slower than clearance from the large airways. The slow clearance phase has now been included in the revised dosimetric model for the human respiratory tract adopted by the ICRP [6], representing clearance of particles deposited in the bronchiolar region.

Primary ciliary dyskinesia (PCD) is an inherited autosomal recessive disorder, characterized by absent or impaired mucociliary transport due to ciliary movement dysfunction. The disorder is clinically manifested by chronic respiratory tract infections beginning in early childhood, leading to chronic bronchitis, chronic rhinosinuitis and otitis media as well as by situs inversus in 50 % of the affected subjects and male infertility [7,8].

Histopathological studies have shown that early morphological changes, i.e signs of an inflammatory process, first appear in the small airways in several lung diseases such as asthma, chronic bronchitis and cystic fibrosis [9,10]. It is not clear which mechanisms contribute to the clearance from the small airways. In an earlier study clearance from small airways was studied up to 96 h, in six subjects with PCD. The retained fraction at 24 h reflecting the large airway was larger compared to that of the healthy controls. A significant clearance occurred in the PCD subjects every 24 h up to 72 h, but after 72 h the clearance declined, but was still faster than alveolar clearance [11]. It is possible that the first 72 h in the PCD subjects reflected clearance from the whole lung, both large, middle and some small sized airways, with a prolonged fast clearance phase representing cough clearance which serves as a compensatory mechanism for their defective MCC. The slower clearance after 72 h probably represents clearance from small airways since it is unlikely that cough can compensate in this region. Even in patients with chronic bronchitis, faster clearance than alveolar clearance occurs from the small airways: [12].

So far, few studies investigating clearance from the small airways have been published since there are limitations in the technique of depositing particles in these airways. One option is to use rather large monodisperse particles (6 μm) and an extremely slow inhalation flow (0.05 L/s), in which the major inhaled fraction will be deposited in the small ciliated airways. A recently performed study in patients with cystic fibrosis has shown that clearance from small airways did not significantly differ from that of the healthy subjects [13]. Studies in PCD patients provide a unique possibility to investigate the clearance mechanism with defective MCC from the small airways.

The aim of this study was to investigate whether long term clearance from the small airways is dependent on normal ciliary function. We defined the small airways using the morphological data from the Weibel model A [14], generations 9–15, corresponding to the terminal bronchioles, which have ciliated epithelium and are less than 2 mm in diameter. The observation period after the first days was used to discriminate between small airway clearance and large airway clearance. Our hypothesis was that clearance from small airways is dependent on normal mucociliary transport.

## Materials and methods

### Subjects and design

Long-term clearance (up to 21 days) from small airways was studied in six non-smoking subjects (two females) with primary ciliary dyskinesia, mean age 23.5 (range: 15–35) yrs, recruited from the paediatric pulmonary department and Stockholm CF-center at Karolinska University Hospital and compared with previous data from ten non-smoking healthy controls [15]. The PCD subjects had clinical and radiological evidence of bronchiectases; three of them had situs inversus totalis. The subjects without situs inversus were examined with nasal or bronchial brush biopsies, and ciliary ultrastructural abnormalities were proven by electron microscopic studies [16]. The PCD subjects were in clinically stable condition during the study, although subj. no 5 completed an i.v. antibiotic treatment at the beginning of the study initiated at signs of low grade infection. There was a significant difference in FEV_1 _and airway resistance between the PCD subjects and the healthy controls (p < 0.05). Otherwise the groups did not differ. Characteristics of the participating subjects and their lung function data are given in table 1.

**Table 1:** Anthropometric and lung function data. Individual data are given for the subjects with PCD and mean data are given for the healthy controls.

Subject no.	Sex	Age(yrs)	Weight(kg)	Height(cm)	Situs inversus	Drug regimen	FVC %pred^a^	FEV_1 _%pred^a^	FEV_1_/FVC %	R_aw_kPa*s*L^-1^
1	F	31	64	175	No	IB	108	94	76	0.143
2	M	15	69	176	Yes	IB OM OA	126	116	84	0.101
3	M	16	47	164	Yes	IB OM OA	98	87	81	0.182
4	F	19	56	168	Yes	Non	92	83	79	0.230
5	M	35	80	183	No	IB IM OM	92	57	51	0.251
6	M	23	83	182	No	IB, IM	94	75	67	0.220

**Mean**		**23.5**	**66.5**	**175**			**102**	**85**	**73,2**	**0.245**
**SD**		**8.3**	**13.8**	**7**			**13**	**20**	**11,4**	**0.073**

Healthy controls	n = 10									

**Mean**	6M/4F	**22.3**	**73.1**	**176**			**109**	**106**	**84,1**	**0.144**

**SD**		**1.9**	**11.7**	**7**			**17**	**13**	**6,8**	**0.032**

M; male, F; female. ^a) ^Predicted values according to Quanjer [20].: IB; inhaled bronchodilatator, IM; inhaled mucolytics, OM; oral mucolytics, OA; profylactic oral antibiotic.

All subjects inhaled 6 μm monodisperse Teflon particles labelled with ^111^In with an extremely slow inhalation flow (ESI), 0.05 L/s, giving deposition mainly in the small airways. Radioactivity over the mouth, throat, lungs and stomach was measured immediately after the inhalation of the test particles. Lung retention was measured at 24 hrs, 7, 14 and 21 days. Correction was made for background activity and physical decay of the radionuclide. To confirm results from our previous study we let three of the PCD subjects (no 1–3) inhale the similar test particles also with normal inhalation flow, 0.5 L/s, giving a more central deposition [17]. This exposure was performed one month after the first exposure. Lung retention was measured at equal time points as the ESI exposure. The regional deposition data were estimated using a model developed at the Karolinska Institutet. In the evaluation of the data, the studied period was divided into two phases. A first fast clearance phase, defined as clearance between 0 and 24 hrs, representing mainly large and medium sized airways, and, a second slow clearance phase, defined as clearance between 24 hrs and day 21, representing mainly small airways. Since clearance after 24 hrs up to one week could include cough clearance from larger airways, a longer study of 7 to 21 days was chosen to represent small airway clearance.

The study was approved by the Ethics Committee of Human Research and the Isotope Committee at the Karolinska University Hospital. All the PCD and the healthy subjects have given their written informed consent to participate in the study. Parental consent was obtained for the subjects younger than 18 years.

### Lung function tests

The pulmonary function was evaluated the same day as the exposure by forced expirograms (Lung Function Laboratory 2100, SensorMedics, Anaheim, CA, USA) giving forced vital capacity (FVC), forced expiratory volume in 1 s (FEV_1.0_), and forced expiratory flow between 25 and 75% of the exhaled volume (FEF_25–75%_). Airway resistance (R_aw_), was measured using a panting technique within a whole-body plethysmograph (Transmural Body Box 2800, SensorMedics). The subjects wore a noseclip and performed the tests in a sitting position. All lung function parameters were determined according to the criteria proposed by Quanjer [20].

### Production and inhalation of test particles

The Teflon particles were produced using the same batch of colloidal Teflon and labelled with ^111^Indium (half-life 68 h) by a spinning disc technique [21]. The reprocessing of the particles before inhalation has been described elsewhere [22]. Mean particle size was 6.2 μm, with a geometric standard deviation of 1.07. The mean aerodynamic diameter was calculated from the density of the Teflon particles, 2.12 g/cm3, as measured by Philipson [21]. The calculated aerodynamic diameter of the Teflon particles was also confirmed by direct measurements of the settling velocity in air [23]. The subjects wore a noseclip and inhaled the particles in a sitting position. The subjects first made a moderately deep exhalation outside the chamber and then inhaled as deep as they could from the chamber. The inhalation flow was measured with a pneumotachograph, placed between the aerosol chamber and the mouthpiece, and was displayed on-line. By looking at the recorder needle, the subjects could inhale at a fairly constant rate throughout the inspiration. All subjects were trained to inhale in this manner before they inhaled the test particles. Between each inhalation from the chamber, the subject could rest and breathe quietly. During the inhalation from the chamber, the PCD subjects had no problem of voluntarily suppressing potential coughs. Measured inhalation flow varied between 0.043–0.049 L/s for all the subjects. The duration of the exposure with slow inhalation flow was on average 6 min and the duration of each breath lasted about 30 s, during which all particles deposited by means of sedimentation. Exhaled radioactivity has earlier been shown to be 0–2% [19]. In the exposure with normal inhalation flow, the flow was measured to 0.45–0.48 L/s and each inhalation included a breath hold of at least 2 sec after full inspiration.

### Measurement of radioactivity

Immediately after inhalation and 24 hours later, the radioactivity was measured using two 127 × 51 mm NaI crystals fitted with collimatorsc. The radioactivity deposited in the lungs was 0.1 MBq. The radioactivity was also measured at 24 hours and at 7 days after inhalation using the whole-body scanner with three large NaI detectors, at the Swedish Radiation Protection Institute (SSI)[4]. The front of each detector facing the subject was provided with focusing slit collimators of lead. The γ-spectra from each detector were acquired separately giving a total of 210 spectra from one measurement. The spectra were later analysed so that the radioactivity in the lung could be distinguished from the activity in the stomach. The technique has been described in detail by Falk et al [18]. At day 7, 14 and 21, when the radioactivity in the gastrointestinal tract was insignificant compared to the lung radioactivity, a more sensitive lung counter with the subject in supine position was used. The relative sensitivity between the lung counter and the whole-lung scanner at 24 h, and between the whole-lung scanner and the low-level lung counter at day 7 was established for each subject by repeated measurements in the two systems within an hour.

Lung retention was measured with the same procedure in the three subjects exposed with normal inhalation flow.

### Statistical analyses

The data were analysed statistically using SPSS 11.0 for Windows. The descriptive data are expressed as mean ± SD and the results as mean (95% confidence interval (CI)) when appropriate. Nonparametric rank sum test for evaluation of differences between the groups and the Spearman correlation coefficient were used; p < 0.05 was considered significant. In the few cases when the measured point differed by 1 day, measurements were adjusted at 2 and 3 weeks using interpolation to day 14 and 21.

## Results

### Deposition

**Figure 1:**
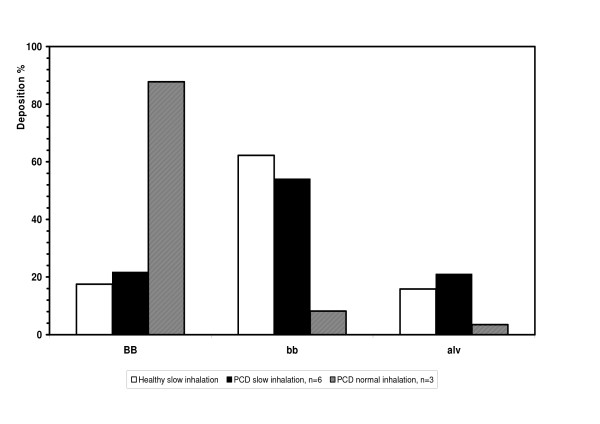
Calculated regional deposition in percentage of total deposition of 6μm particles (D_ae_) inhaled with extremely slow inhalation flow (0.05 L/s) in ten healthy controls □ and six subjects with PCD ■ in large airways (BB, generations 0–8), small airways (bb, generations 9–15) and alveolar region (alv, generations 16–23) according to the airway model proposed by Weibel 14. For comparison 3 PCD subjects inhaled 6μm particles with normal inhalation flow (0.5 L/s) ▨.

The calculated regional deposition in different airway generations with the extremely slow inhalation flow, according to the theoretical model, showed that the main fraction was deposited in the small airways (in generations 9–15: 54–62%), i.e the bronchiolar region. Calculation for the PCD subjects and the healthy controls showed similar deposition pattern with only a small fraction (16–22 %) deposited in the alveolar region. With normal inhalation flow a substantially larger fraction of the particles in the three subjects exposed was deposited in the large airways (mean 74%, mouth and throat excluded). With normal inhalation flow only 14% of total deposition was calculated to be deposited in the small airways, Figure 1.

### Fast airway clearance

**Figure 2:**
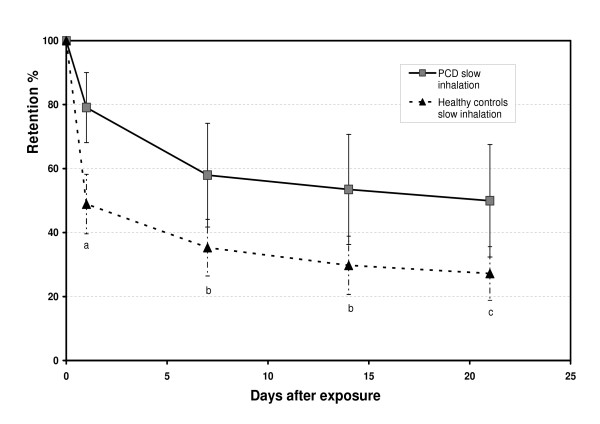
The mean retention of 6 μm particles (D_ae_) in percentage of deposition in six subjects with PCD and ten healthy controls using an extremely slow inhalation flow, 0.05 L/s. The bars show the SD of the mean. Significant difference ^a) ^p < 0.001, ^b) ^p < 0.005, ^c) ^p < 0.01 comparing retention between the two studied groups.

A significant larger lung retention at 24 h in percentage of the initial lung deposition (Ret_24_) was found in the PCD subjects, 79 % (95% CI 67.6;90.6), compared to the healthy subjects, 49 % (42.3;55.5), p < 0.001. A difference in lung retention compared to the healthy subjects persisted through out the study (p < 0.005) to the last measured point at day 21, Table 2 and Figure 2.

**Table 2:** Particle retention in percent of deposition (mean, ± SD) at 24 h, 7, 14 and 21 days after exposure.

	All PCD n = 6	Healthy controls n = 10	Normal flow 0.5 l/s n = 3	Slow flow 0.05 l/s n = 3
Ret_24h_	79.1 ± 10.9*	48.9 ± 9.3*	68.6 ± 7.5	73.9 ± 1.6
Ret_7d_	57.9 ± 16.2**	35.3 ± 8.8**	42.1 ± 8.7	50.8 ± 6.9
Ret_14d_	53.5 ± 17.2**	29.7 ± 9.1**	40.2 ± 8.7	45.5 ± 6.5
Ret_21d_	49.9 ± 17.6**	27.2 ± 8.4**	38.1 ± 8.6	41.2 ± 7.4

*: p < 0.001 ** p < 0.01 for the PCD patients versus reference data from healthy controls using Mann-Whitney rank sum test.

### Slow airway clearance

**Figure 3:**
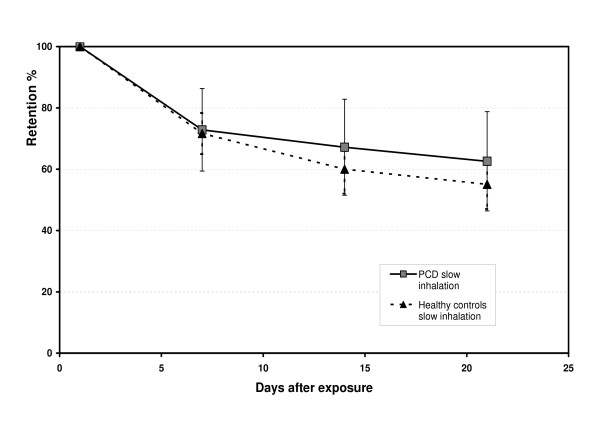
The mean retention of 6 μm particles (D_ae_) in percentage of retention at 24 hrs in six subjects with PCD and ten healthy controls, using an extremely slow inhalation flow, 0.05 L/s. The bars show the SD of the mean.

Both in the PCD subjects and in the healthy controls there was significant difference in retention between each time point indicating a significant measured clearance continuing in the small airways in both groups. Overall, the slow clearance phase between day 1 and day 21 did not differ significantly between the PCD subjects and the healthy controls (Figure 3). There was no correlation between FEV_1 _and R_aw _with Ret_24 _or clearance between day 7 to day 21. Also the subject who completed an i.v. antibiotic treatment during the study did not differ significant from the average clearance pattern, e.g. was not in the outer range.

**Figure 4:**
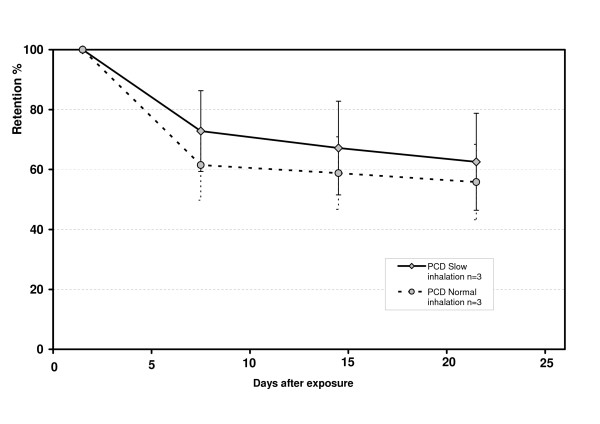
The mean retention of 6 μm particles (D_ae_) in percentage of retention at 24 h in three of the PCD subjects with extremely slow inhalation flow, 0.05 L/s (full drawn line), and with normal flow, 0.5 L/s (dotted line). The bars show the SD of the mean.

The Ret_24 _was not significantly larger for particles inhaled at 0.05 L/s, (75, 75 and 72%) compared to 0.5 L/s (76, 61, 69%) in the three PCD subjects (p = 0.28). With normal flow, 0.5 L/s, only 9% of the fraction retained at day 7 was cleared the following two weeks, compared to 19% with the extremely slow inhalation flow, Figure 4.

## Discussion

Clearance in the small airways is difficult to study, most of all since deposition in this region is difficult to target. The objective of this study was to investigate long-term clearance from small airways in subjects with a known ciliary dysfunction and to compare their clearance data with data from healthy controls. The design of the study with PCD subjects studied over a longer period was 1) to select a population with known defective ciliary function and 2) to ascertain that clearance from large to middle size airways was completed.

## Deposition

In this study we used rather large particles inhaled with extremely slow inhalation to maximize deposition in the small airways. The advantage of the method is that regional deposition is independent in respect to airway constriction, since the extremely slow inhalation flow decreases impaction and the deposition is mainly due to sedimentation [24]. Extremely slow inhalation probably also reduces deposition by impaction in areas with bronchiectases. The predicted lung deposition of the inhaled particles was to a large extent in the small airways. Our model for regional deposition predicted very small differences between the PCD subjects and the healthy controls (Figure 1), even if there was a difference in FEV_1 _and airway resistance between the PCD subjects and the healthy controls. The significantly larger retention day 1 (Ret_24_) in the PCD subjects compared to the healthy controls (Figure 2) also indicates deposition mainly in the small airways, a result which is in agreement with our earlier study in PCD subjects [11]. Particles deposited more centrally in the lung would be effectively cleared from large airways by coughing, resulting in a smaller difference of the Ret_24 _compared to the healthy controls [11,17]. If the fraction of particles instead had been to a larger extent deposited in the alveolar region, the clearance after 24 h would have been much slower than what was found. The alveolar clearance of insoluble particles (similar to ours) has been shown to be very slow and may take years to complete [25]. We therefore conclude that the particles were deposited to a larger extent in the small airways as suggested from estimates using the model developed at the Karolinska Institutet [4,5,18,19] both in the PCD subjects as well as in the healthy controls.

### Fast airway clearance

Normally, the fast airway clearance is concluded within 24 h in healthy subjects shown in studies both with extremely slow and normal inhalation techniques [1]. For 4.2 μm aerodynamic diameter ferromagnetic iron oxide particles inhaled with the shallow bolus technique, approximately 50 % of the deposited fraction clears by the fast mucociliary clearance mechanism with a mean clearance half life of 3.0 ± 1.6 h [26]. Our data shows the same result among the healthy subjects, with a cleared fraction of 51% within 24 h (Table 2 and Fig 2). However, in the PCD subjects there was a substantial fraction retained at 24 h (Ret_24_), 79.1 ± 10.9, showing that the fast airway clearance was prolonged due to impaired mucociliary clearance depending on ciliary dysfunction. Similar findings have been reported by Regnis et al measuring mucociliary clearance with gamma scintigraphy up to 24 h in PCD patients [27]. In the present study the fast clearance phase continued in the PCD subjects up to one week (Table 2, Fig 2). Similar result was also found by our group in patients with CF, using the same design as in this study [13]. Also the recently published study by Möller et al [28], measuring clearance up to 270 days using ferromagnetic particles inhaled with the shallow bolus technique, indicated a prolonged fast clearance phase measured at 1, 2 and 7 days in the PCD patients. In the earlier study with the same type of particles and slow inhalation technique by Svartengren et al [11], the clearance among PCD subjects measured every 24 h up to 96 h showed that the fast clearance phase was prolonged up to 72 h : Even if the patients with CF and PCD, of our studies, have daily inhalations and physiotherapy the voluntary coughing could only partially compensate for their defective MCC. To establish an effective cough clearance, sufficient high velocity of airflow is needed, which can only be obtained in the larger tracheobronchial region approximately down to generation 7, together with a productive mucus production [29,30].

The impaired MCC, predominantly affecting the fast clearance phase, probably has impact on the progression of the disease. After being diagnosed of PCD and with the introduction of a treatment program, most of the patients lung function is stabilized [31]. This shows that appropriate use of antibiotics and mucus dissolving agents, together with effective airway clearance techniques, e.g. physiotherapy, is effective enough to keep the fast clearance phase within the basic needs. Despite the apparent similarities with CF, PCD has a much better prognosis. The progression of the lung damage in CF is probably due to the mucus properties with reduced local immunological response rather than the mucociliary movement dysfunction [32].

### Slow airway clearance

In healthy subjects, the slow clearance phase, using the shallow bolus deposition technique, was shown to have a half life of 109 ± 78 days measured over a period of 9 month [26]. However, some of the ferromagnetic particles in the study could have reached the alveolar region and the result may represent both small airways and alveolar clearance. Previous clearance studies using the extremely slow inhalation technique and particle size as in this study, in both healthy subjects and various groups of patients, have shown a slow clearance phase continuing for several weeks with kinetics faster than alveolar but also clearly different from large airway clearance [25,33].

The present study focused on clearance from the small airways in patients with a known ciliary dysfunction (PCD), investigating whether normal mucociliary transport is necessary for clearance in the small airways. The results show that the mean cleared fraction between 24 h and 7 days in percent of the available fraction at 24 h was 27% in the PCD subjects and 28% in the healthy controls. The small airway clearance was equivalent for the PCD subjects and the healthy controls, using either day 1 or day 7 as starting point for the slow airways clearance up to the last measured point at 21 days (Figure 3). In support to our findings, the study by Möller et al showed that after the prolonged fast clearance phase was concluded, there was no difference in the fraction of long-term retained particles between the PCD patients and the healthy subjects [28]. The PCD subjects ultrastructual abnormalities have been shown to correlate to low cilia activity scores. [34] Even if the PCD subjects may have some ciliary movements, our results suggest that MCC is less important in the small airways and that other mechanisms than MCC apparently are present and probably responsible for a substantial clearance. This hypothesis is also supported by a study using adrenergic stimulation. Normally, terbutaline stimulates MCC [35], but no increased clearance from small airways was found in healthy controls when pre-stimulating MCC by oral intake of terbutaline and then inhaling the test particles [36]. All but one of the PCD subjects were treated with brochodilatator during the study. The subject (no 4) with no treatment had a retained fraction at 24 h of 98 %, subsequently 15 % of the retained particles at 24 h cleared within 21 days. It is possible that MCC stimulating agents together with cough clearance are most important for the fast clearance phase, representing the larger airways. In the smaller airways the airflow is much slower due to the large cross-sectional area and consequently cough clearance is less effective. But cough could, as a mechanical force, influence other secondary mechanisms in the small airways. The only stimulating effect on clearance from small airways documented so far is continuous positive airway pressure therapy (CPAP) in children with Duchennes muscular dystrophy [37]. Increasing age has also been shown to have a negative association to slow airway clearance measured between 1–21days [15].

The clearance is highly dependent on where the particles deposit; in large airways; small airways or in the alveolar region. With normal inhalation flow, the particles deposit partly in the larger airways and partly in the alveolar non-ciliated region. This difference between normal and slow inhalation flow is illustrated in the three subjects who inhaled the test particles also with normal inhalation flow. A slightly lower Ret_24 _(69%) was shown using the normal inhalation flow, compared to the slow inhalation flow (74%), in the three subjects.

With more centrally deposition the fast clearance phase, representing above all cough clearance in the PCD subjects, continued after 24 h with an average cleared fraction, between 24 h and day 7, of 39% of Ret_24_. After day 7 the clearance turned very slow, representing mainly alveolar clearance, and the average cleared fraction between day 7 to day 21, in percentage of remained fraction day 7, was only 9% compared to 19% with slow inhalation flow (Figure 4).

Other possible clearance mechanisms besides MCC in the small airways could be phagocytosis by airway macrophages or penetration to the sub-mucus space between the cilia. Airway macrophages are rapidly recruited to the sites of the particle deposition and phagocytose the particles [38]. The more loaded the macrophages are, the more rapidly they disappear from the conducting airways [39]. In vivo particle uptake by airway macrophages in healthy subjects showed a maximal uptake at 100 min after aerosol inhalation suggesting that a resident population of macrophages is present on the airway surface [40]. If this really reflects the macrophage action in small airways has however not been verified since the sampling of the particles and macrophages in this study was with induced sputum method derived from the larger airways [41]. The thickness of the mucous layer varies by location in the conducting airways, and has been reported to be 8.3 μm in the trachea to about 1.8 μm in the small bronchioles. There are evidence that the mucus layer in the small bronchi and bronchioles is consisting of discontinues patches rather than a continuous layer [42]. The particles are coated with surfactant and then submerge and penetrate to the aqueous layer between the cilia where they can be engulfed both by macrophages and by dentritic cells [43]. Penetration of particles through the epithelium is probably more important for smaller particles than those used in the present study, although new evidence indicates limited translocation and elimination from the lung also for ultrafine particles [44].

## Conclusion

The purpose of this study was to investigate if mucociliary transport as a mechanism has a significant importance for the clearance in the small airways. Subjects with primary ciliary dyskinesia were chosen since they have a known ciliary dysfunction. Even though the number of included PCD subjects in this study was small, it was shown that rather large particles, inhaled with an extremely slow inhalation flow, resulted in increased deposition in small airways equal to the healthy controls according to the model calculation. The larger deposited fraction in the small airways, however, resulted in larger lung retention at 24 h and a prolonged fast clearance phase in the PCD subjects due to their defective MCC and could not be compensated by cough clearance. The slow airway clearance after 24 h continued faster than alveolar clearance despite their defective ciliary function. The clearance from small airways in the PCD subjects, measured as lung retention day 1–21, had similar clearance rate as the healthy controls. This study does not support the hypothesis that clearance from small airways is dependent on normal ciliary function only. There are apparently additional mechanisms acting in the small airways. The small airways that constitute of a transitional zone between the tracheobronchial and the alveolar region still remain an unknown area. Further investigation about other possible clearance mechanisms acting in the small airways is needed.

## Competing interests

The author(s) declare that they have no competing interests.

## Authors' contributions

ML was the principal investigator, performed the study and the main preparation of the manuscript. RF and KP performed the production of the particles and were responsible for measurements of the radioactivity. MS contributed to the study design, the evaluation of the data and the preparation of the manuscript. LH contributed with the clinical investigation and the preparation of the manuscript. All authors have read the manuscript and accept it in the present form.
